# KBTBD7 promotes non‐small cell lung carcinoma progression by enhancing ubiquitin‐dependent degradation of PTEN

**DOI:** 10.1002/cam4.4794

**Published:** 2022-05-02

**Authors:** Zifang Zou, Bo Zhang, Zhihan Li, Lei Lei, Guanghao Sun, Xizi Jiang, Jingqian Guan, Yao Zhang, Shun Xu, Qingchang Li

**Affiliations:** ^1^ Department of Thoracic Surgery The First Hospital of China Medical University Shenyang People's Republic of China; ^2^ Department of Pathology First Affiliated Hospital of Dalian Medical University Dalian People's Republic of China; ^3^ Department of Pathology The Second Hospital of Dalian Medical University Dalian People's Republic of China; ^4^ Department of Pathology The First Hospital of China Medical University Shenyang People's Republic of China

**Keywords:** epidermal growth factor, invasion, non‐small cell lung cancer, proliferation, PTEN, the Kelch repeat and BTB domain containing 7

## Abstract

The Kelch repeat and BTB domain containing 7 (KBTBD7) was first cloned in 2010. Its function as a transcriptional activator and a substrate adaptor during the ubiquitination process was soon found. KBTBD7 was shown to be involved in excessive inflammation after myocardial infarction, brain development, and neurofibromin stability. However, studies on the role of KBTBD7 in solid tumors, especially lung cancer, are still lacking. Therefore, in this study, we investigate the role of KBTBD7 in non‐small cell lung cancer (NSCLC). Immunohistochemical staining of 104 paired NSCLC and peritumoral normal specimens indicated that KBTBD7 was highly expressed in NSCLC tissues and positively correlated with the histological type, P‐TNM stage, lymph node metastasis, and tumor size. KBTBD7 was also well‐expressed in NSCLC cell lines, and downregulation of KBTBD7 resulted in inhibition of NSCLC cell proliferation and invasion. Further investigation showed that KBTBD7 enhanced ubiquitin‐dependent degradation of PTEN, thus activating EGFR/PI3K/AKT signaling and promoting NSCLC cell proliferation and invasion by regulating CCNE1, CDK4, P27, ZEB‐1, Claudin‐1, ROCK1, MMP‐9, and E‐cadherin protein levels. Our results indicate that KBTBD7 may be a potential therapeutic target for the treatment of NSCLC.

## Graphical abstract

KBTBD7 was highly expressed in NSCLC tissues and positively correlated with the P‐TNM stage, lymph node metastasis, and tumor size.KBTBD7 enhanced ubiquitin‐dependent degradation of PTEN, thus activating EGFR/PI3K/AKT signaling and promoting NSCLC cell proliferation and invasion by regulating CCNE1, CDK4, P27, ZEB‐1, Claudin‐1, ROCK1, MMP‐9, and E‐cadherin protein levels.

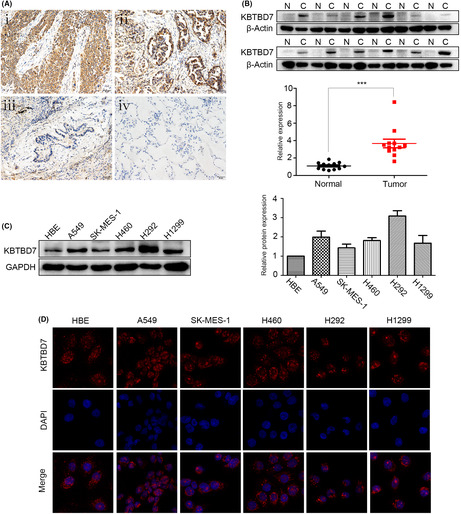

## 1 INTRODUCTION

Lung cancer remains the leading cause of cancer‐related deaths; 1.8 million people died of lung cancer worldwide in 2020, representing 18% of all cancer deaths.^1^ Based on the current situation, the incidence of lung cancer will continue to increase over the decades, especially in developing countries. In recent years, people have been paying more attention to health check‐ups due to the advent of detection modalities, such as low‐dose CT. However, most patients are still diagnosed at an advanced stage, which is not operable. The survival rate of lung cancer at 5 years after diagnosis is only 10%–20% globally, based on the latest data.^1^ The characteristics of lung cancer are changing, and during our clinical practice in Northeast China, we found that the proportion of adenocarcinoma has increased rapidly in the past decade. The incidence rates among women are also increasing, possibly due to air pollution and exposure to smoke from cooking.^2^, ^3^ Thus, we urgently need to discover new oncogenes and suppressor genes that target the constantly changing lung cancer and provide patients with more treatment options.

The Kelch repeat and BTB domain containing 7 (KBTBD7) was first cloned from a human embryonic heart complementary DNA library containing the BTB and Kelch domains.^4^ Researchers soon found that it could induce serum response element (SRE) and activator protein‐1 (AP‐1)‐mediated transcriptional activation.^4^ KBTBD7 is also the substrate adaptor of CUL3, which mediates ubiquitin–proteasome degradation. KBTBD7 participates in the degradation of T‐lymphoma and metastasis gene 1 (TIAM1) and dopamine type 2 receptor (DRD2) proteins, leading to the restriction of TIAM1‐RAC1 signaling and enhancing the dopamine agonist resistance to pituitary adenoma, respectively.^5^, ^6^ In 2018, KBTBD7 was shown to promote inflammatory responses in macrophages, and microRNA‐21 can directly target KBTBD7 to prevent cardiac dysfunction and inflammation after myocardial infarction.^7^ However, research on KBTBD7 in lung cancer is still lacking.

EGFR/PI3K/AKT signaling plays a vital role in the occurrence and development of lung cancer, it regulates cancer cell apoptosis, proliferation, migration, and differentiation.^8^ PTEN is an important tumor suppressor that exhibits dual lipid and protein phosphatase activities. PTEN mutations have been implicated in several types of tumors, such as breast, thyroid, prostate, melanoma, and lung.^9^, ^10^ In lung cancer, decreased levels of PTEN were correlated with an advanced stage of lung cancer.^11^ PTEN dephosphorylates PIP3 back to PIP2, which opposes the activity of PI3K/AKT signaling. In the past decade, many studies have reported that PTEN could directly bind to EGFR and accelerate the downregulation of activated EGFR, thus suppressing EGFR/PI3K/AKT signaling.^12^, ^13^

In this study, we explore the expression of KBTBD7 in NSCLC specimens and aim to verify the underlying mechanism of KBTBD7 regulating non‐small cell lung carcinoma (NSCLC) cell phenotype.

## 2 METHODS

### 2.1 Patients and specimens

In total, 104 paired NSCLC and peritumoral normal specimens were collected from patients with NSCLC who underwent surgery at the Department of Thoracic Surgery of the First Hospital of China Medical University from 2018 to 2020. None of the patients underwent chemotherapy or radiotherapy before surgery. Written informed consent was obtained from each patient, and the study was approved by the Medical Research Ethics Committee of China Medical University.

### 2.2 Immunohistochemistry and immunofluorescence

For immunohistochemistry, the collected tissue specimens were serially sliced at a thickness of 4 μm. Specimens were kept at 70°C for 4–6 h before the immunohistochemical staining. After xylene deparaffinization and gradient alcohol hydration, the slices were repaired at a high temperature in a slightly boiling ethylenediaminetetraacetic acid (EDTA) repair solution for 20 minutes. Immunohistochemistry was performed using IHC kits (MaixinBio). The primary antibody used in the experiment was anti‐KBTBD7 rabbit antibody (1:100; NBP3‐05059; Novus). The immunohistochemical scoring standards were as follows: KBTBD7 staining intensity was categorized as 0 (no staining), 1 (weak staining, light yellow), 2 (medium staining, yellow), or 3 (strong staining, dark yellow or brown). The KBTBD7‐stained area was categorized as 1 (1%–25%), 2 (26%–50%), 3 (51%–75%), or 4 (76%–100%). The two scores were multiplied for each specimen to obtain a final score of 0–12. Specimens with scores >6 were considered KBTBD7‐positive, whereas those with scores ≤6 were considered KBTBD7‐negative. Immunofluorescence assays were performed as previously described.^14^

### 2.3 Cell culture and transfection

NSCLC cell lines were purchased from the Cell Bank of the China Academy of Sciences. Human bronchial epithelial (HBE) cells were obtained from the American Type Culture Collection. All cells were cultured in a medium containing 10% fetal bovine serum (FBS) and placed in a 5% carbon dioxide incubator at 37°C. Transfection was performed using Lipofectamine 3000 reagent (Invitrogen) according to the manufacturer's instructions. The shRNA plasmids shKBTBD7 and sh‐NC were purchased from RiboBio. PTEN‐specific small interfering RNA (siRNA) and negative control siRNA were also purchased from RiboBio. The sequences were as follows: shKBTBD7, GTATGATAGGGAAGAT and siPTEN, GGTGTAATGATATGTGCAT.

### 2.4 Western blot and co‐immunoprecipitation

Western blot assays were performed as previously described.^15^ For co‐immunoprecipitation assays, cells were extracted using NP40 (Beyotime Biosciences, Jiangsu, China) containing 1% PMSF (Beyotime Biosciences) and 1% phosphatase inhibitor cocktail (Biotool). The lysate was immunoprecipitated with anti‐PTEN antibodies (#9188, CST) or control anti‐IgG rabbit antibodies (A7016; Beyotime Biosciences). The immunocomplex was then captured using ProteinA+G agarose beads (P2012; Beyotime Biosciences). After boiling for 10 min, the immunocomplexes were dissociated from the beads and analyzed by Western blot.

### 2.5 CCK‐8 and colony formation assays

The assays were performed as previously described.^16^ For CCK‐8 assay, 3000 cells were placed into each well of a 96‐well plate containing 100 μl of medium, and the absorbance was measured at 450 nm using a microplate reader for 5 days. For the colony formation assay, 800 cells were placed into each 60‐mm cell culture dish containing 4 ml of medium. The cells were fixed and stained after 10–14 days of incubation.

### 2.6 Transwell invasion assay

Invasion assays were performed using a 24‐well transwell chamber with a pore size of 8 μm (Costar Group), and the inserts were coated with 100 μl of Matrigel (1:9 dilution, BD Bioscience). For A549 or H1299 cells, 1 × 10^4^ or 8 × 10^3^ cells, respectively, were placed in the upper chamber with 200 μl of medium containing 2% FBS and the lower chamber with 800 μl of medium containing 20% FBS. The cells were fixed with polyformaldehyde and stained with hematoxylin after 24 hours of incubation.

### 2.7 RNA extraction and RT‐PCR

The assays were performed as previously described.^17^ The primer sequences were as follows: KBTBD7, forward 5′‐AGACGCCTTCGACCATCAC‐3′ and reverse 5‐GAATTGAACCCATTCGGCTGA‐3; PTEN, forward 5′‐TGGATTCGACTTAGACTTGACCT‐3′ and reverse 5′‐GGTGGGTTATGGTCTTCAAAAGG‐3′; EGFR, forward 5′‐GGAGAACTGCCAGAAACTGACC‐3′ and reverse 5′‐GCCTACAGCACACTGGTTG‐3′; GAPDH, forward 5′‐AGACGCCTTCGACCATCAC‐3′ and reverse 5‐GAATTGAACCCATTCGGCTGA‐3.

### 2.8 Ubiquitination assays and immunoprecipitation

The assays were performed as previously described.^18^ The stable KBTBD7 knockdown NSCLC cells were transfected with Ub‐HA plasmid, and then pre‐treated with MG‐132 (HY‐13259, MCE) for 12 hours before collection. After immunoprecipitation with anti‐PTEN (#9188, Cell Signaling Technology), the level of PTEN ubiquitination was evaluated using anti‐HA immunoblotting (HT301, TransGen Bio) by Western blot.

### 2.9 Statistical analysis

Statistical analysis was achieved using the SPSS 17.0 and GraphPad Prism 6.0 software. The chi‐squared test was implemented for evaluating the correlation between KBTBD7 expression and clinicopathological parameters. Comparisons between two groups were analyzed by paired Student's *t*‐tests. Statistical significance was set at *p* < 0.05.

## 3 RESULTS

### 3.1 KBTBD7 is highly expressed in NSCLC tissues and correlates with clinicopathological characteristics

**FIGURE 1:**
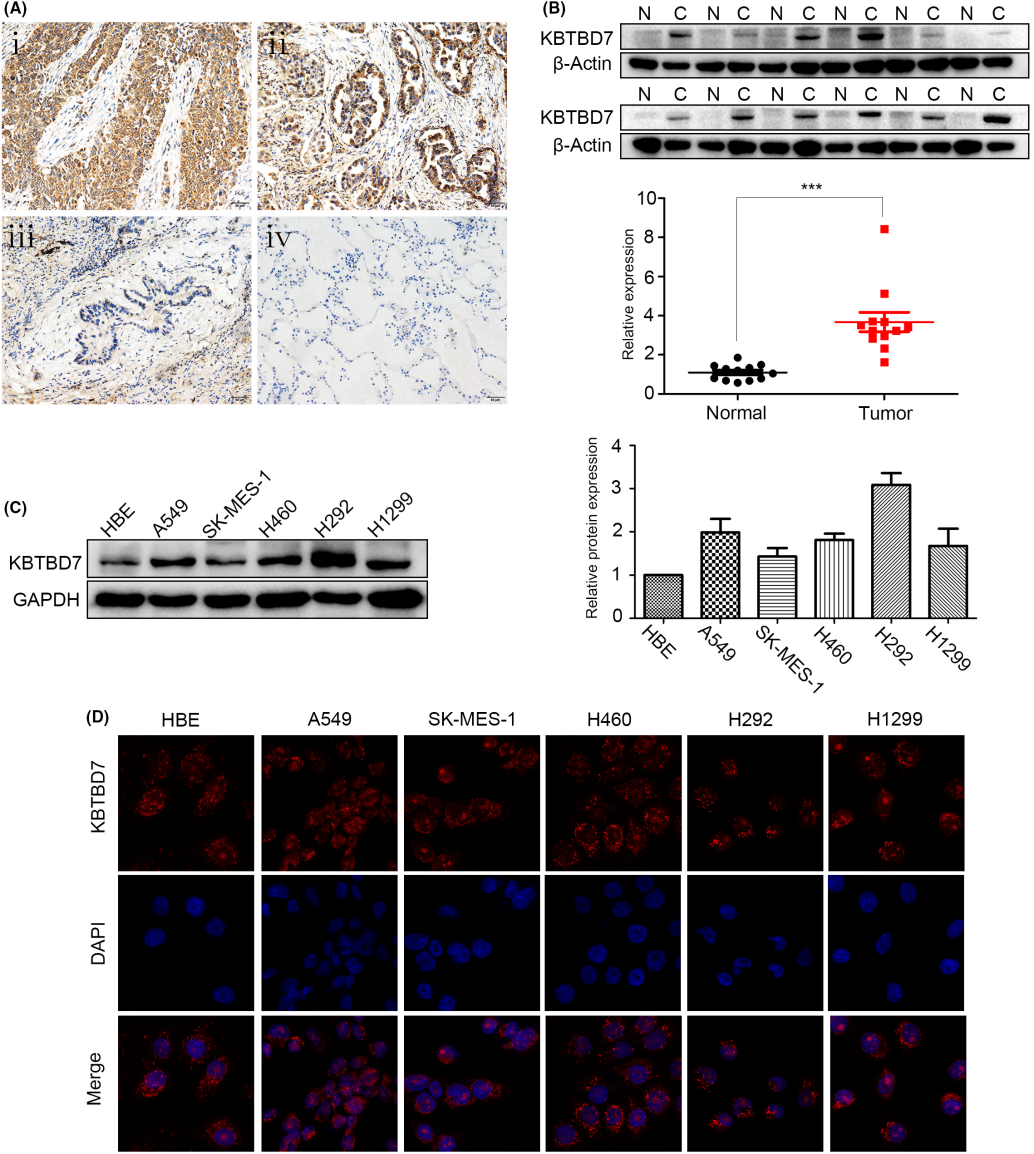
KBTBD7 is highly expressed in NSCLC tissues and cell lines. (A) KBTBD7 expression is positive in NSCLC tissues: squamous carcinoma (i) and adenocarcinoma (ii) but negative in paired normal bronchial (iii) and alveolar epithelial cells (iv). Magnification, ×200. (B) Western blot results show that KBTBD7 is highly expressed in fresh NSCLC tissues (C) compared to that in corresponding noncancerous tissues (N). Relative quantification of protein expression was analyzed by the Image J software. *p < 0.05; **p < 0.01. (C) Western blot results indicate that the KBTBD7 protein level is increased in A549, SK ‐MES ‐1, H460, H292, and H1299 cells compared to that in HBE cells. Relative quantification of protein expression was analyzed by the Image J software. (D) Immunofluorescence assays indicate that KBTBD7 is located in the cytoplasm of NSCLC cell lines. Magnification, ×400.

KBTBD7 expression in 104 paired NSCLC and adjacent non‐cancerous tissues was assessed by immunohistochemical staining. The results indicated that KBTBD7 was strongly expressed in the cytoplasm of NSCLC specimens compared to paired normal specimens (Figure 1A). Statistical analysis revealed that KBTBD7 overexpression was positively correlated with histological type (*p* = 0.028), p‐TNM stage (*p* = 0.019), lymph node metastasis (*p* = 0.034), and tumor size (*p* < 0.01) (Table 1). We also collected 14 paired fresh NSCLC and matched non‐cancerous specimens. Western blot results indicated that KBTBD7 was overexpressed in NSCLC specimens contrasted to matched non‐cancerous specimens (Figure 1B).

**TABLE 1:** Correlation of KBTBD7 expression with clinicopathological parameters of NSCLC patients

Clinicopathological characteristics	Total *N*	KBTBD7‐negative	KBTBD7‐positive	*p*‐value
Age (years)				
≤60	53	16	37	0.484
>60	49	18	31	
Gender				
Male	72	27	45	0.167
Female	30	7	23	
Histological type				
Squamous cell carcinoma	52	22	30	0.028
Adenocarcinoma	50	11	39	
Differentiation				
Well‐moderate	61	19	42	0.568
Poor	41	15	26	
Tumor size (cm)				
≤3	37	19	18	<0.01
>3	65	15	50	
Lymph node metastasis				
Negative	52	14	36	0.034
Positive	50	24	28	
TNM stage				
I–IIA	45	22	23	0.019
IIB–III	57	15	42	

### 3.2 KBTBD7 expression and subcellular localization in NSCLC cell lines

After verification in NSCLC specimens, KBTBD7 expression was then explored in NSCLC cell lines. KBTBD7 protein levels were assessed in six types of NSCLC cell lines and HBE cells. Western blot assays indicated that KBTBD7 was expressed at high levels in SK, A549, H1975, H1299, and HCC827 cell lines compared to the HBE cell line (Figure 1C). Immunofluorescence assays were also performed to verify the localization of KBTBD7 in NSCLC and HBE cell lines, and it showed that KBTBD7 was localized in the cytoplasm of these cells (Figure 1D).

### 3.3 Suppressing KBTBD7 inhibits the proliferation and invasion of NSCLC cells

**FIGURE 2:**
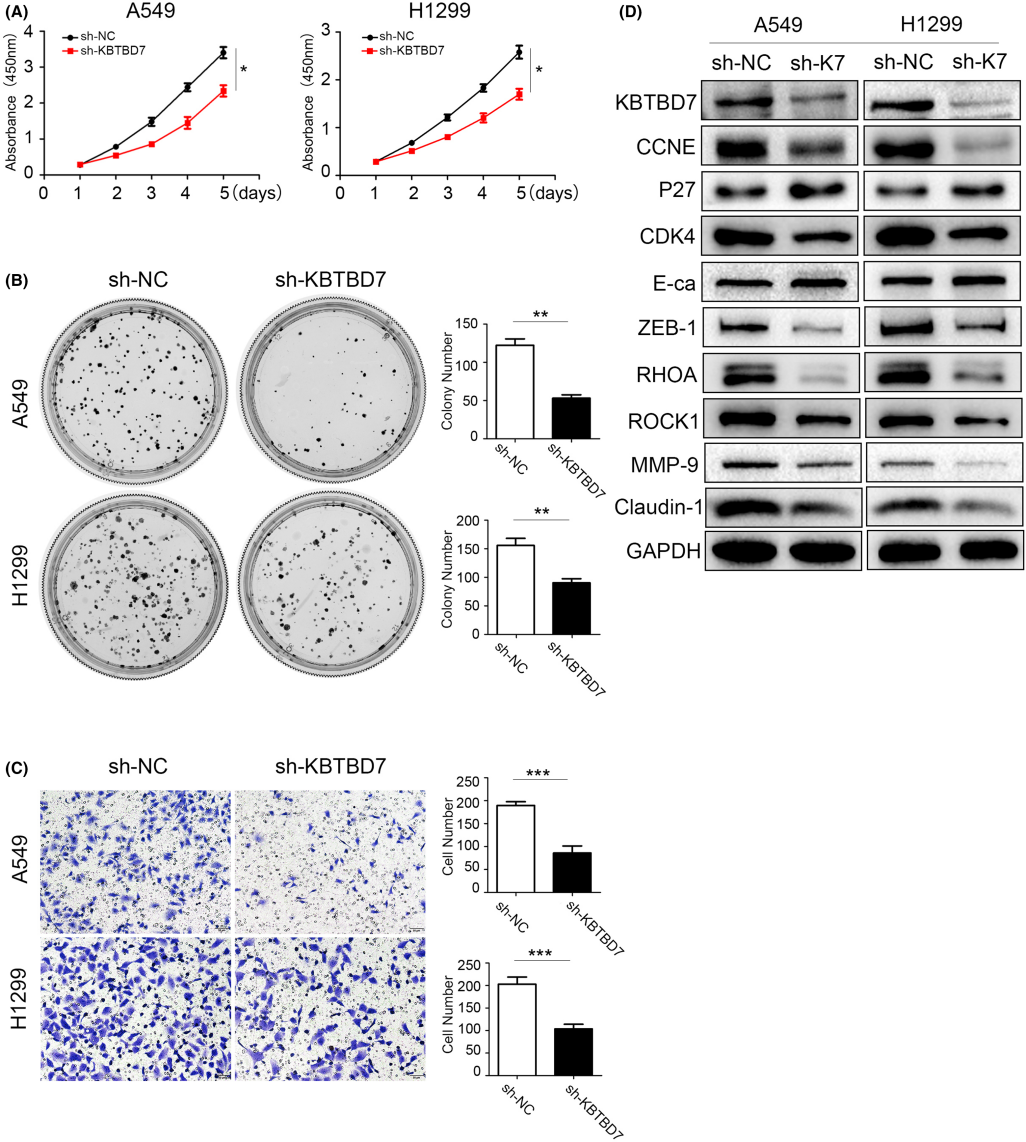
Suppressing KBTBD7 inhibits NSCLC cell proliferation and invasion. (A, B) CCK‐8 and colony formation assays indicate that A549 and H1299 cell proliferation is suppressed when KBTBD7 is knocked down. *p < 0.05; **p < 0.01. (C) Transwell assays indicate that invasion of A549 and H1299 cells is inhibited when KBTBD7 is suppressed. *p < 0.05; **p < 0.01. (D) CCNE, CDK4, ZEB‐1, Claudin‐1, Rock1, and MMP‐9 are downregulated when KBTBD7 (K7) is knocked down; E‐cadherin and P27 protein levels are upregulated when KBTBD7 (K7) is knocked down.

Based on the results that KBTBD7 is overexpressed in NSCLC cell lines, we suppressed KBTBD7 in A549 and H1299 cells to explore whether KBTBD7 regulates the phenotype of NSCLC cells. CCK‐8 and colony formation assays revealed that downregulating KBTBD7 significantly inhibited the proliferation and colony formation abilities of NSCLC cells (Figure 2A,B). Transwell assays indicated that suppressing KBTBD7 significantly inhibited the invasion ability of NSCLC cells (Figure 2C). The expression of proliferation‐related proteins was then examined by western blot and the results showed that CCNE1 and CDK4 were downregulated while P27 was upregulated when KBTBD7 was suppressed (Figure 2D). We next detected the expression of invasion‐related proteins. The results showed that MMP‐9, Claudin‐1, Rock1, and ZEB‐1 were downregulated while E‐cadherin was upregulated when KBTBD7 was suppressed (Figure 2D).

### 3.4 KBTBD7 influences EGFR/PI3K/AKT signaling via regulating PTEN

**FIGURE 3:**
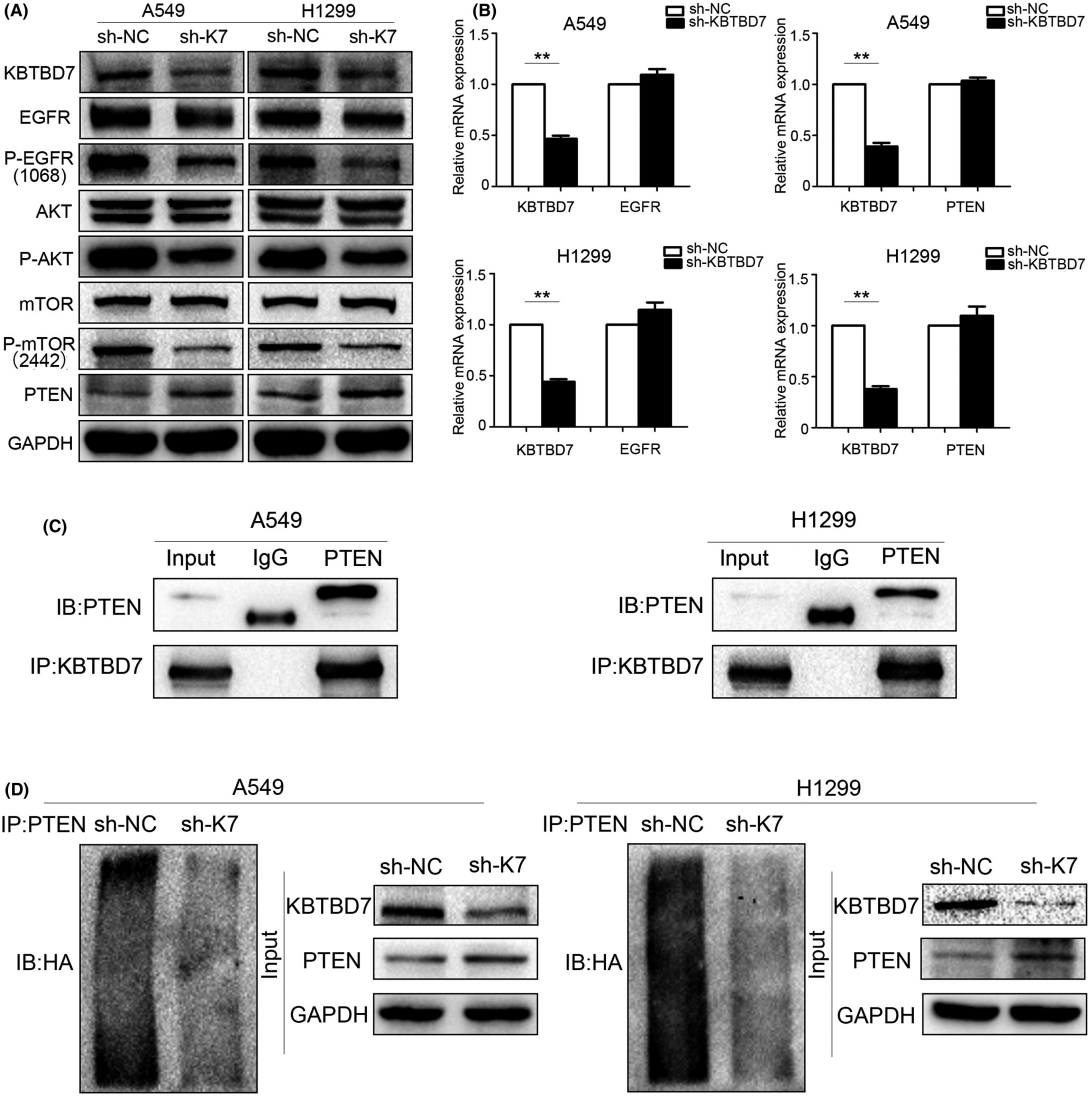
KBTBD7 interacts with PTEN and increases ubiquitin‐dependent degradation of PTEN. (A) Elevated EGFR, p‐EGFR (1068), AKT, p‐AKT (473), mTOR, p‐mTOR (2442), and PTEN protein levels are detected when KBTBD7 (K7) is suppressed in A549 and H1299 cells. (B) EGFR and PTEN mRNA are detected when KBTBD7 is knocked down in A549 and H1299 cells. (C) Interaction between KBTBD7 and PTEN in A549 and H1299 cells was verified by co‐immunoprecipitation assays. (D) PTEN ubiquitination is detected when KBTBD7 (K7)is knocked down in A549 and H1299 cells. KBTBD7 suppressed cells were transfected with HA‐ubiquitin plasmid and then immunoprecipitated by the anti‐PTEN antibody.

To further explore the mechanism by which KBTBD7 regulates the biological functions of NSCLC cells, we performed western blot to examine the changes in the key proteins of some vital signaling pathways. The results indicated that the protein levels of EGFR, p‐EGFR (1068), p‐AKT (473), and p‐mTOR (2448) decreased significantly when KBTBD7 was knocked down in NSCLC cells (Figure 3A). RT‐PCR assays were then conducted to detect the mRNA levels of EGFR. The results showed that there was no obvious change in EGFR mRNA levels when KBTBD7 was knocked down, indicating that KBTBD7 regulates EGFR and EGFR signaling at the post‐translational level (Figure 3B). Co‐immunoprecipitation assays were performed to detect protein interactions between KBTBD7 and EGFR, but the results were negative. Thus, we found that KBTBD7 influenced EGFR signaling, and the regulation was indirect.

**FIGURE 4:**
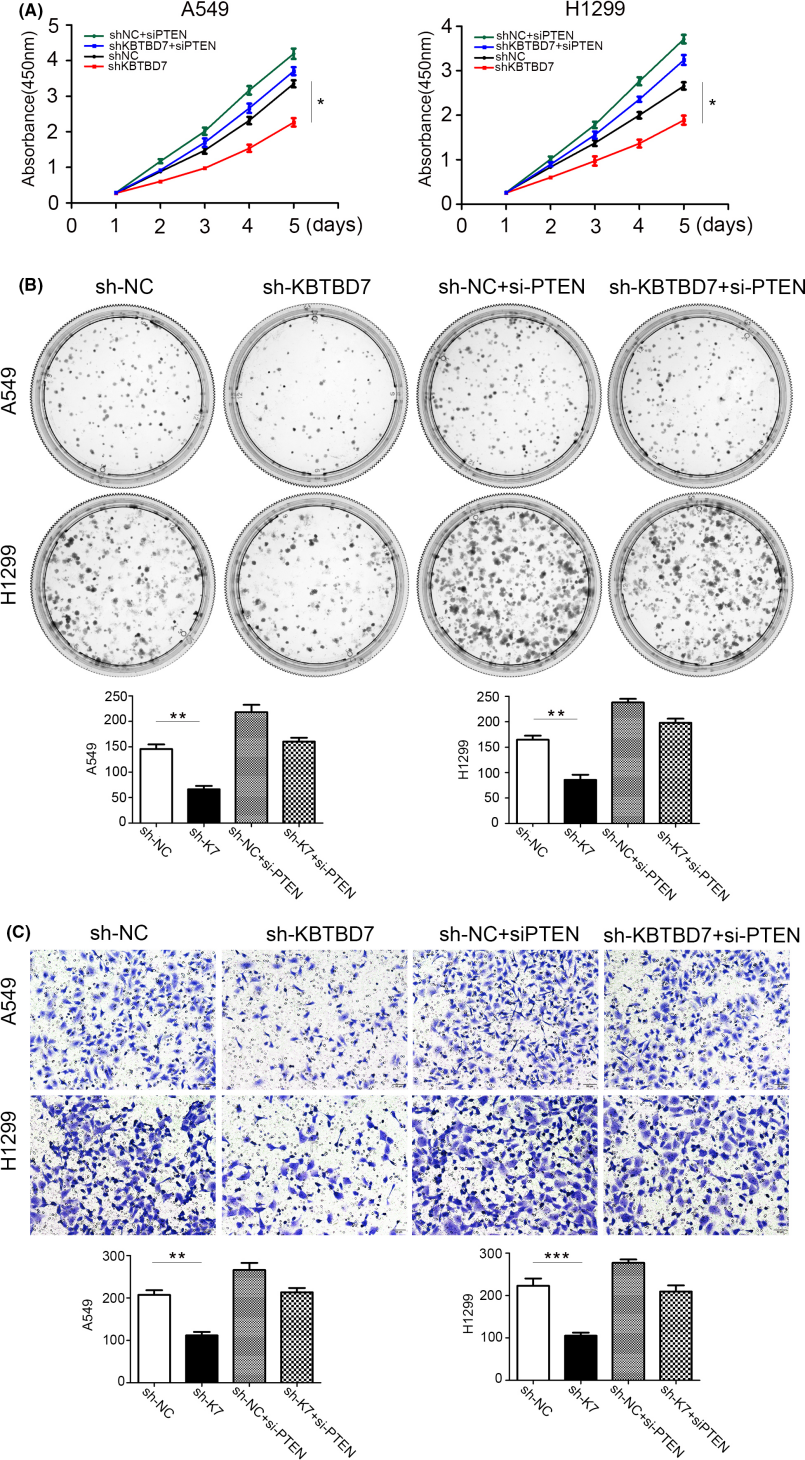
KBTBD7 influences the proliferation and invasion of NSCLC cells via PTEN regulation. (A, B) CCK‐8 and colony formation assays show that the changes in A549 and H1299 cell proliferation induced by suppression of KBTBD7 are rescued by siPTEN. *p < 0.05; **p < 0.01. (C) Transwell assays show that the changes in A549 and H1299 cell invasion induced by suppression of KBTBD7 are rescued by siPTEN. *p < 0.05; **p < 0.01.

**FIGURE 5:**
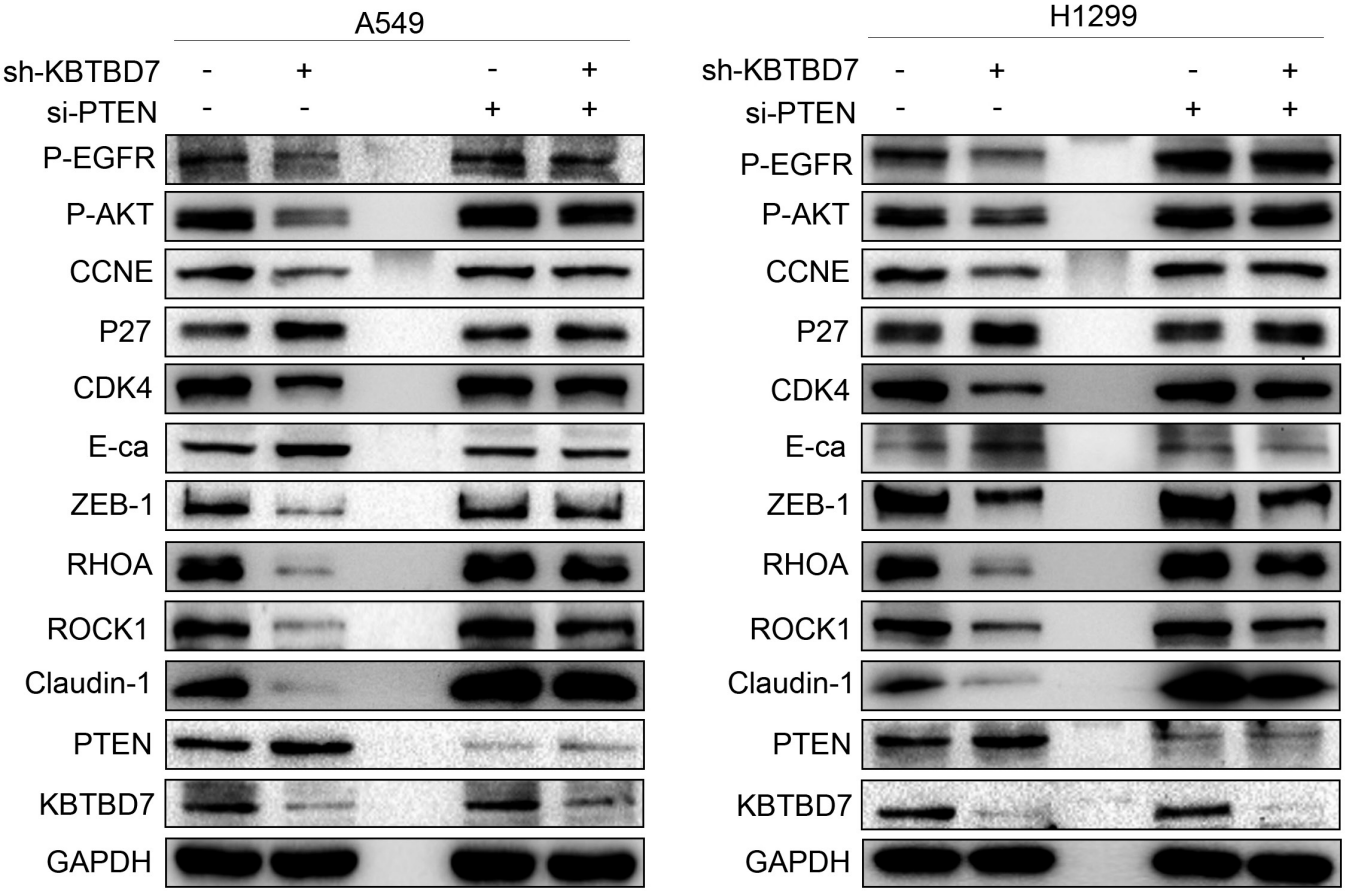
KBTBD7 influences proliferation and invasion of NSCLC cells via PTEN regulation. The changes in pEGFR(1068), p‐AKT(473), p‐mTOR(2442), CCNE, CDK4, P27, ZEB‐1, Claudin‐1, Rock1, E‐cadherin, and MMP‐9 levels by suppressing KBTBD7 in A549 and H1299 cells are rescued by siPTEN.

PTEN is a tumor suppressor implicated in various kinds of malignant tumors. Several studies have verified that PTEN regulates EGFR and EGFR/PI3K/AKT signaling by accelerating ligand‐induced EGFR degradation. Given the aforementioned effect of KBTBD7 on EGFR and EGFR signaling, we examined whether KBTBD7 also regulated the PTEN expression. Western blot results showed that PTEN was upregulated when KBTBD7 was suppressed in NSCLC cells (Figure 3A). As mentioned above, we hypothesized that KBTBD7 affects EGFR/PI3K/AKT signaling and NSCLC phenotype by regulating the expression of PTEN. To confirm this, we used siRNA to knock down PTEN and determine if siPTEN could rescue the changes in NSCLC cells caused by KBTBD7 suppression. CCK‐8, colony formation, and Transwell invasion assays showed that siPTEN reduced the inhibition of cell proliferation and invasion (Figure 4A–C). The changes in p‐EGFR, p‐AKT, p‐mTOR, CCNE1, CDK4, P27, ZEB‐1, Claudin‐1, Rock1, E‐cadherin, and MMP‐9 induced by KBTBD7 suppression were also rescued by siPTEN (Figure 5). These data suggest that the influence of KBTBD7 on EGFR/PI3K/AKT signaling and the biological functions of NSCLC cells depend on PTEN expression.

### 3.5 KBTBD7 interacts with PTEN and increases ubiquitin‐dependent degradation of PTEN

RT‐PCR analysis showed no change in PTEN mRNA levels when KBTBD7 was suppressed in NSCLC cells (Figure 3B). Therefore, the regulatory role of KBTBD7 on PTEN may occur at the post‐translational level rather than at the mRNA level. Co‐immunoprecipitation assays verified that KBTBD7 interacts with PTEN at the protein level (Figure 3C). We suspected that KBTBD7 may influence the degradation process of PTEN. For confirmation, ubiquitination assays were performed. Cells were pre‐treated with MG132 for 12 h before collection. The ubiquitination of PTEN was evaluated by immunoprecipitation using an anti‐PTEN antibody, followed by anti‐HA immunoblotting. The results indicated that KBTBD7 enhanced ubiquitin‐dependent degradation of PTEN (Figure 3D), as expected.

## 4 DISCUSSION

In this study, we found that KBTBD7 was highly expressed in NSCLC tissues and positively correlated with histological type, differentiation, the P‐TNM stage, lymph node metastasis, and tumor size. Suppressing KBTBD7 inhibited the proliferation and invasion of NSCLC cells by regulating CCNE1, CDK4, P27, ZEB‐1, Claudin‐1, Rock1, MMP‐9, and E‐cadherin protein levels. Further experiments showed that KBTBD7 influenced EGFR/PI3K/AKT signaling activity. As KBTBD7 affect EGFR amount but not EGFR mRNA level, we assumed that KBTBD7 might interact with EGFR and regulate its stability. But the co‐immunoprecipitation result was negative, which means we still need to find the key protein by which KBTBD7 regulate EGFR signaling.

As a well‐known tumor suppressor, PTEN regulates cancer cell angiogenesis, motility, genomic stability, proliferation, and the tumor microenvironment.^19^, ^20^ Besides dephosphorylating the lipid second messenger PIP3, PTEN has also been reported to be associated with EGFR stability. Several studies have verified that PTEN stabilizes the EGFR–CBL complex and promotes EGFR degradation^13^; PTEN could also facilitate EGFR trafficking to late endosomes by dephosphorylating Rab7; thus, enhancing EGFR endocytic trafficking.^21^ During our research, we found that KBTBD7 regulates PTEN expression, and further rescue assays showed that KBTBD7’s influence on EGFR/PI3K/AKT signaling depends on PTEN expression. We next explored the mechanism of KBTBD7 regulating PTEN amount. The expression and function of PTEN is regulated by various modifications, including phosphorylation, oxidation, ubiquitination, and acetylation.^19^ Alterations in PTEN ubiquitination may cause disordered protein metabolism and induce tumorigenicity of cancer cells.^22^, ^23^, ^24^ As a substrate adaptor of CUL3, KBTBD7 participates in the ubiquitin–proteasome degradation process. Co‐immunoprecipitation and ubiquitination assays verified that KBTBD7 enhances ubiquitin‐dependent degradation of PTEN, as expected. The NSCLC and peritumoral normal specimens we used during immunohistochemistry assays were obtained from patients who underwent surgery from 2018 to 2020, so we are unable to collect the prognostic information by now, which is a limitation of our research.

EGFR‐targeted therapy is getting more and more important during clinical practice treating NSCLC patients, both as an initial stand‐alone treatment or as an adjuvant treatment after surgical resection. EGFR sensitive mutations are identified in approximately 10%–50% of NSCLC patients worldwide, with the Asian ethnicity having the highest mutation frequency.^25^ EGFR‐tyrosine kinase inhibitor (TKI) showed higher objective response rates and progression‐free survival when used in the treatment of advanced NSCLC patients compared to traditional cytotoxic therapy.^26^ However, primary and acquired resistance remain the main obstacles to the long‐term efficacy of EGFR‐TKI therapy. Plenty of research has proven that PTEN loss or deficiency contributes to EGFR‐TKI resistance, which is an independent predictor of EGFR‐TKI treatment outcome.^27^ Since our results verified that KBTBD7 influences EGFR/PI3K/AKT signaling by regulating PTEN ubiquitin‐dependent degradation, we hypothesize that KBTBD7 may also be related to the EGFR‐TKI treatment response and could be a potential therapeutic target for NSCLC.

## AUTHOR CONTRIBUTIONS

ZZF, ZB, XS, and LQC conceived the research. ZZF conducted the experiments and wrote the manuscript. ZB and LZH contribute to the cell culture and proliferation assays. LL and SGH contribute to Western blot and RT‐PCR assays. JXZ and GJQ contribute to Immunohistochemistry assays. ZY contributes to the statistical analysis.

## CONFLICT OF INTEREST

The authors confirm that there is no conflict of interest.

## Data Availability

The data used to support the findings of this study are available from the corresponding author upon request.
