# Correction to “KBTBD7 promotes non‐small cell lung carcinoma progression by enhancing ubiquitin‐dependent degradation of PTEN”

**DOI:** 10.1002/cam4.7462

**Published:** 2024-09-09

Zou Z, Zhang B, Li Z, Lei L, Sun G, Jiang X, Guan J, Zhang Y, Xu S, Li Q. KBTBD7 promotes non‐small cell lung carcinoma progression by enhancing ubiquitin‐dependent degradation of PTEN. *Cancer Med*. 2022 Dec;11(23):4544–4554.

In the Abstract section, the text “Immunohistochemical staining of 104 paired NSCLC and peritumoral normal specimens indicated that KBTBD7 was highly expressed in NSCLC tissues and positively correlated with the histological type, P‐TNM stage, lymph node metastasis, and tumor size” was incorrect. It should read “Immunohistochemical staining of 102 paired NSCLC and peritumoral normal specimens indicated that KBTBD7 was highly expressed in NSCLC tissues and positively correlated with the histological type, P‐TNM stage, lymph node metastasis, and tumor size.”

In the Method section, 2.1 Patients and specimens “In total, 104 paired NSCLC and peritumoral normal specimens were collected from patients with NSCLC who underwent surgery at the Department of Thoracic Surgery of the First Hospital of China Medical University from 2018 to 2020.” was incorrect. It should read “In total, 102 paired NSCLC and peritumoral normal specimens were collected from patients with NSCLC who underwent surgery at the Department of Thoracic Surgery of the First Hospital of China Medical University from 2018 to 2020.”

In the Result section, 3.1, “KBTBD7 expression in 104 paired NSCLC and adjacent non‐cancerous tissues was assessed by immunohistochemical staining.” was incorrect. It should read “KBTBD7 expression in 102 paired NSCLC and adjacent noncancerous tissues was assessed by immunohistochemical staining.”

In the Result section, 3.2, “Western blot assays indicated that KBTBD7 was expressed at high levels in SK, A549, H1975, H1299, and HCC827 cell lines compared to the HBE cell line (Figure 1C).” was incorrect. It should read “Western blot assays indicated that KBTBD7 was expressed at high levels in A549, SK, H460, H292, and H1299 cell lines compared to the HBE cell line (Figure 1C).”

Figure 3 was incorrect. The correct figure is shown here:

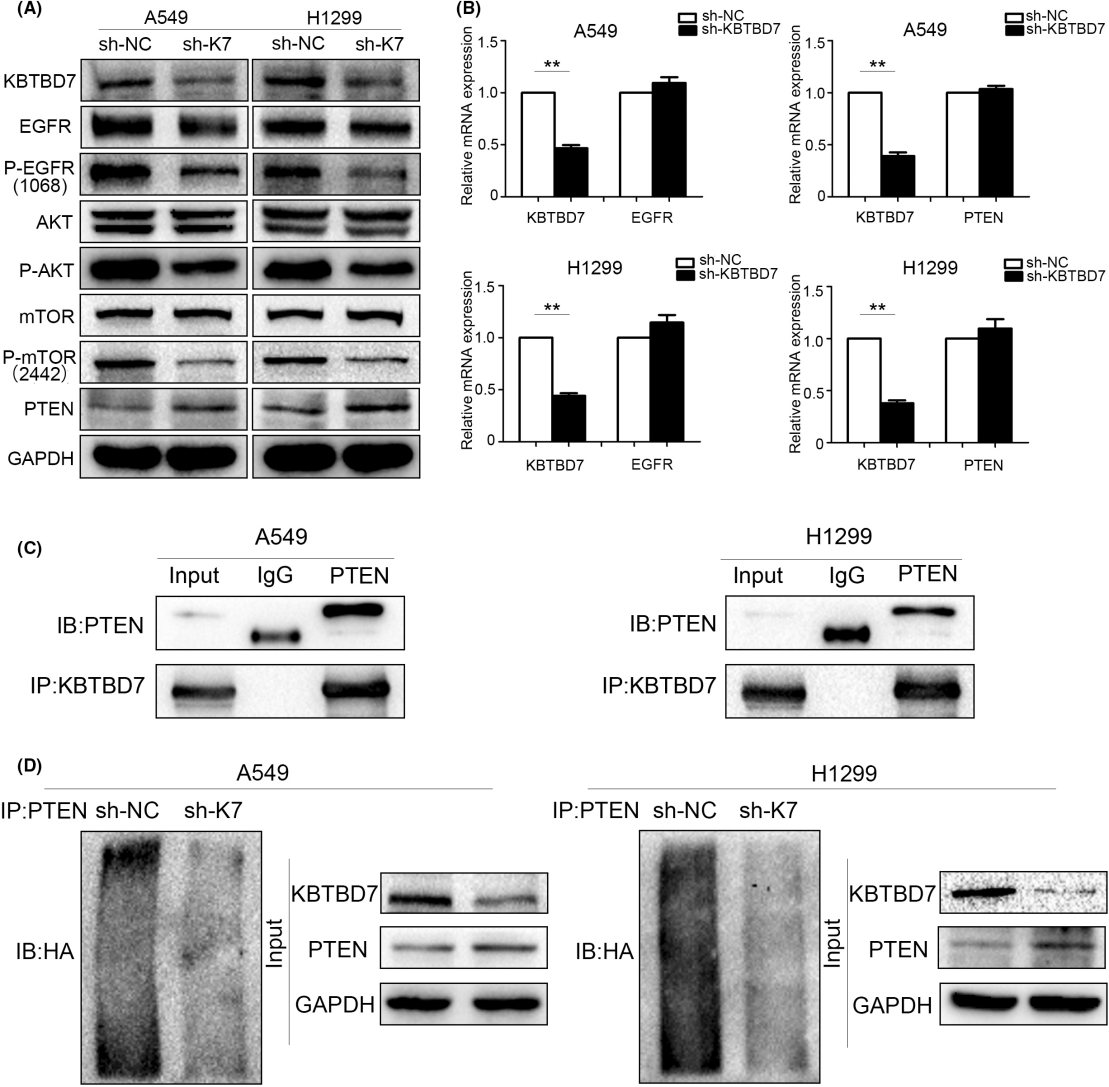

Table 1 was incorrect. The correct table is shown here:

**TABLE 1:** Correlation of KBTBD7 expression with clinicopathological parameters of NSCLC patients.

Clinicopathological characteristics	Total *N*	KBTBD7‐negative	KBTBD7‐positive	*p*‐Value
Age (years)				
≤60	53	16	37	0.484
>60	49	18	31	
Gender				
Male	72	27	45	0.167
Female	30	7	23	
Histological type				
Squamous cell carcinoma	52	22	30	0.028
Adenocarcinoma	50	11	39	
Differentiation				
Well‐moderate	61	19	42	0.568
Poor	41	15	26	
Tumor size (cm)				
≤3	37	19	18	<0.01
>3	65	15	50	
Lymph node metastasis				
Negative	52	24	28	0.034
Positive	50	14	36	
TNM stage				
I–IIA	45	22	23	0.019
IIB–III	57	15	42	

We apologize for these errors.

